# Quantitative sequence-function relationships in proteins based on gene ontology

**DOI:** 10.1186/1471-2105-8-294

**Published:** 2007-08-08

**Authors:** Vineet Sangar, Daniel J Blankenberg, Naomi Altman, Arthur M Lesk

**Affiliations:** 1 Department of Biochemistry and Molecular Biology, Center of Computational Biology and Genomics, The Huck Institute for Genomics, Proteomics and Bioinformatics, The Pennsylvania State University, University Park, PA 16802, USA; 2 Department of Statistics, The Pennsylvania State University, University Park, PA 16802, USA

## Abstract

**Background:**

The relationship between divergence of amino-acid sequence and divergence of function among homologous proteins is complex. The assumption that homologs share function – the basis of transfer of annotations in databases – must therefore be regarded with caution. Here, we present a quantitative study of sequence and function divergence, based on the Gene Ontology classification of function. We determined the relationship between sequence divergence and function divergence in 6828 protein families from the PFAM database. Within families there is a broad range of sequence similarity from very closely related proteins – for instance, orthologs in different mammals – to very distantly-related proteins at the limit of reliable recognition of homology.

**Results:**

We correlated the divergence in sequences determined from pairwise alignments, and the divergence in function determined by path lengths in the Gene Ontology graph, taking into account the fact that many proteins have multiple functions. Our results show that, among homologous proteins, the proportion of divergent functions decreases dramatically above a threshold of sequence similarity at about 50% residue identity. For proteins with more than 50% residue identity, transfer of annotation between homologs will lead to an erroneous attribution with a totally dissimilar function in fewer than 6% of cases. This means that for very similar proteins (about 50 % identical residues) the chance of completely incorrect annotation is low; however, because of the phenomenon of recruitment, it is still non-zero.

**Conclusion:**

Our results describe general features of the evolution of protein function, and serve as a guide to the reliability of annotation transfer, based on the closeness of the relationship between a new protein and its nearest annotated relative.

## 1. Background

Assignment of function to gene products in the absence of direct experimental information is an important challenge of computational molecular biology [1-3]. In annotating proteins from newly-sequenced genomes, it is a common practice to transfer functional annotation from a homologous protein [4-8]. This approach depends on the assumptions that: (1) because homologous proteins have similar sequences and structures, they have similar functions, and (2) the annotation of the source homologue is correct. Often, but certainly not always, these assumptions are valid.

In this study we quantitatively assess the relationship between the divergence of protein function and the divergence of amino acid sequence in families of homologous proteins. In addition to illuminating the process by which proteins evolve altered and novel functions, the results provide guidance about the expected accuracy of transfer of functional annotation among homologous proteins in databases.

The most general evidence for protein homology, and inference of shared function, depends on comparative analysis of sequences and structures. PSI-BLAST [9] and Hidden Markov Models [10] identify distant homologs from multiple sequence alignments. Other techniques include the training of support vector machines [11] and neural networks [12] on protein features such as charge distribution and hydrophobicity to predict protein function. Structure comparisons improve the accuracy of inference of function in the absence of direct experimental evidence. These include the use of information from domains [13] and motifs [14-16]. Fleming *et al*. [17] combined structural and sequence alignments of proteins in an annotation tool named PHUNCTIONER.

Despite the sensitivity of these tools for detecting homologs and predicting function, many authors have pointed out that because closely-related proteins can change function, either through divergence to a related function or by recruitment for a very different function, annotations based only on homology can be incorrect [18-28].

Two problems that have arisen in studying the evolution of protein function and evaluating the expected accuracy of functional annotation transfer have been (1) standardization of terminology in describing function, and (2) defining a measure of the "distance" between functions. The Enzyme Commission classification has been very valuable but deals with only one class of protein functions [29]. In 2000, The Gene Ontology (GO) Consortium formulated a newer and more general classification of protein functions and the relationships among them [30]. Unlike the EC classification, which was a strict hierarchy, the GO scheme has the form of Directed Acyclic Graphs (DAGs), specialized to three domains: Molecular Function, Biological Process, and Cellular Component.

Enzyme Commission identifiers form a strict four-level hierarchy, or tree. For example, isopentenyl-diphosphate Δ-isomerase is assigned EC number 5.3.3.2, where the initial 5 specifies the most general category, 5 = isomerases; 5.3 comprises intramolecular isomerases; 5.3.3 those enzymes that transpose C = C bonds; and the full identifier 5.3.3.2 specifies a particular reaction. Note that the EC classified *reactions*, not enzymes. To compare functional assignments of two proteins according to the EC classification, it is conventional to ask at how many levels of the hierarchy the EC numbers agree.

In contrast, the GO classification is not a tree, but a more general type of graph. Each node is labeled by a general or specific protein function. Edges in the graph correspond to relationships between more general and more specific functions, that is, child-parent relationships. For example, the node "protein binding" is a child of the node containing the more general function "binding". The number of levels – the length of the path from any leaf to the root – is not constant. The structure of the GO DAG induces a measure of distances between functions, which will be used to quantify sequence-function relationships in proteins (see Materials and Methods).

**Figure 1:**
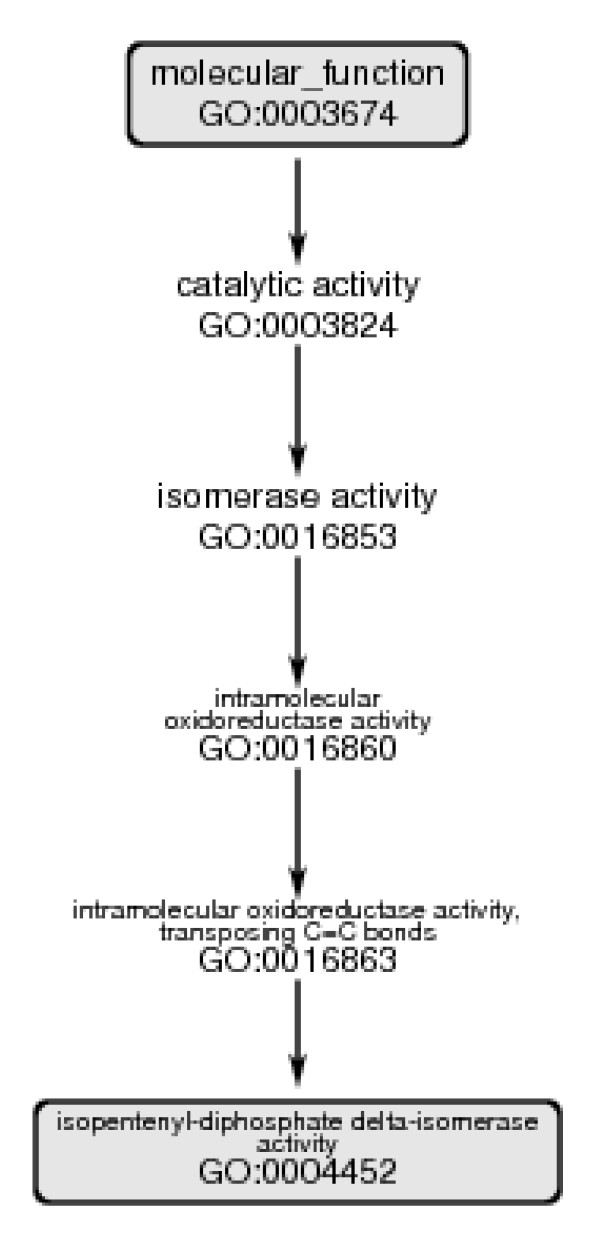
The minimal-length path from GO:0004452 to the root node in the molecular function ontology.

GO assigns the identifier 0004452 to isopentenyl-diphosphate Δ-isomerase. (The numbers themselves have no specific significance.) Figure 1 shows a minimal-length path from GO:0004452 to the root node of the molecular function DAG, GO:0003674. In this case there are four intervening nodes, progressively more general categories as we move up the figure. Note that the GO description of this enzyme as an oxidoreductase is inconsistent with the EC classification, in which a committed choice between oxidoreductase and isomerase must be made at the highest level of the EC hierarchy.

Our current work treats the Molecular Function component of the GO classification. The GO Molecular Function graph forms a network that has characteristics in common with other biological networks. In the Gene Ontology DAGs, the average in-degree is 1.36 (that is, on average a node or GO ID had 1.36 parents.) The in-degree distribution is intermediate between an exponential and a power function. There is a wide range in out-degree, ranging from 1 to 298. Three nodes had very high out-degree with 122, 238 and 298 children. The out-degree distribution followed a power law, showing that there are hubs, or highly connected nodes. The total degree (in-degree + out-degree) distribution for the Molecular Function ontology has a mean of 2.69, and follows a power law.

### 1.1 Assignment of functions to proteins

Neither the Enzyme Commission nor the GO classifications of protein function constitutes an assignment of function to any particular protein. Both provide only a framework for making such assignments. The PIR database at Georgetown University [31] associates Gene Ontology Identifiers (GO IDs) with individual proteins. The annotation of each protein may include several GO IDs. Indeed, annotation with any function logically implies annotation with all more-inclusive functions, all the way up to the root of the graph. (Note, however, that annotations of proteins by GO terms in databases do not always explicitly contain all the ancestors of every function that appears.) Therefore for each protein we extracted the *distal *(= most precise) GO IDs to represent the function of the protein (see Materials and Methods).

### 1.2 The relationship between sequence divergence and function divergence

Many proteins with similar sequences have similar functions; for example, mammalian hemoglobins transport oxygen and carbon dioxide. For mammalian hemoglobins, transfer of annotation among homologs gives correct results. However, other families of homologs contain proteins with different functions. For example, hen egg white lysozyme and baboon α-lactalbumin have 37% identical residues in optimal sequence alignment, and retain very similar mainchain structures, but have unrelated functions. Contrasting mammalian hemoglobins with lysozyme/α-lactalbumin, there is a general correlation between divergence of sequence and divergence of function. That is, mammalian hemoglobins have similar sequences and similar functions; lysozyme and α-lactalbumin have more distantly related sequences and dissimilar functions.

However, there are many exceptions to this correlation. In the duck, eye lens crystallins are identical in sequence to liver enolase and lactate dehydrogenase [32]. This is an example of "recruitment" – unrelated function with little or even no sequence change. This threatens to produce incomplete or even erroneous annotations, if annotation is passed freely among homologs. Conversely, some proteins very distantly related in sequence nevertheless retain similar function.

Several groups have studied the relationship between sequence similarity and functional similarity based on the Enzyme Commission classification. Those studies were necessarily limited to proteins with enzymatic functions:

In studying the relationship between sequences and EC classifications of proteins, Wilson, Kreychman & Gerstein [33], Todd, Orengo & Thornton [34], and Devos & Valencia [19] reached similar (although not identical) optimistic conclusions. Wilson, Kreychman & Gerstein [33] concluded that for pairs of single-domain proteins, at levels of sequence identity > 40%, precise function is conserved, and for levels of sequence identity > 25%, broad functional class is conserved (according to a functional classification that uses the EC hierarchy for enzymes, and supplements it with material from FLYBASE [35] for non-enzymes.) The study of Todd, Orengo & Thornton [34] analyzed only the homologous pairs of enzymes and reported that approximately 90% of pairs of proteins with sequence identity > 40% conserve all four EC numbers. Even at 30% sequence identity, Todd, Orengo & Thornton found conservation of three levels of the EC hierarchy for 70% of homologous pairs of enzymes. Devos & Valencia [19] reached very similar conclusions; they also reported the ability to predict correctly the agreement of FSSP categories [36] and SWISS-PROT [37] keywords, as a function of the level of sequence similarity.

Our work pursues the question of the relationship between divergence of sequence and function in homologous proteins, using the Molecular Function DAG of Gene Ontology for the classification of function. Use of the GO classification allows extension of the earlier work to proteins with non-enzymatic functions, permitting a comprehensive study of functions of proteins.

The steps of our analyses were as follows: For each pair of homologous proteins from a PFAM family, we recorded the % identical residues in the optimal alignment as a measure of sequence divergence, and we measured the functional distance between the sets of distal GO IDs associated with the two proteins. We based our definition of the distance between sets of annotations on a generalization of the simple minimum-path-length measure of the distance between two single GO ID's (see Materials and Methods).

**Figure 2:**
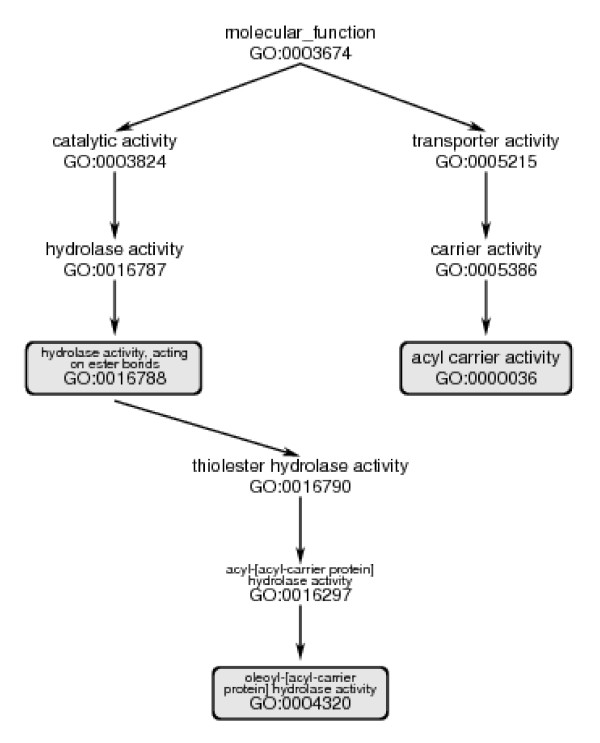
Distinction between similar and dissimilar function. We regard *hydrolase activity*, *acting on ester bonds *and *oleoyl-[acyl-carrier protein] hydrolase activity*, as similar functions, because their lowest common ancestor, *hydrolase activity*, *acting on ester bonds*, is not the root node of the molecular function DAG. However, we would regard *hydrolase activity*, *acting on ester bonds *and *acyl carrier activity*, as dissimilar functions, because their lowest common ancestor is the root node of the DAG. The Figure also illustrates the idea of the distal GO IDs that we extract from an annotation set, in this case describing proteins in the Acyl_ACP thioesterase family. Both *acyl carrier activity *and *oleoyl-[acyl-carrier protein] hydrolase activity *have no child nodes within the GO molecular function DAG. These annotations are therefore as specific as possible within the GO function classification. That is, they are distal both within the annotations of this family of proteins and in the overall GO DAG itself. The third GO ID, *hydrolase activity*, *acting on ester bonds*, annotates some proteins that are *not *annotated with the more precise function *oleoyl-[acyl-carrier protein] hydrolase activity*. For such proteins, *hydrolase activity*, *acting on ester bonds *is a distal GO ID.

From these data, we mapped the relationship between sequence divergence and function divergence. We distinguished divergence of functions within the same "branch" of the DAG (those for which the lowest common ancestor of two nodes was not the root node) and those in different "branches" of the DAG. We call these *similar *and *dissimilar *functions, respectively (Figure 2). We observed that, despite counterexamples of recruitment, there is a general correlation between divergence of sequence and appearance of dissimilar functions within each family. This relationship is made precise by our calculations. Our results also show that there is some variation among different PFAM families, especially for more highly-diverged sequences.

## 2. Results and Discussion

We analyzed 6828 PFAM families (out of a total of 7863 in v. 18.0). The families ranged widely in size, from 2 to > 1200 proteins. Most families were relatively small; 85% of those studied had between 2 and 30 members.

For each pair of proteins within each family we determined the sequence similarity and the set of minimum distances between distal GO IDs (see Materials and Methods). For the functional distances, we distinguished, and analyzed separately, divergent functions within the same branch of the GO DAG (which we call similar functions); and entirely different functions, for which the root of DAG was the lowest common ancestor (which we call dissimilar functions).

A primary goal is to describe the relationship between sequence divergence and functional divergence.

### 2.1 The EF-hand family

The EF-hand family is typical and provides illustrative results. This family contains 498 proteins comprising two classes of functions: signaling and buffering/transport. EF-hand proteins involved in signaling include the best-known members of the family such as calmodulin, troponin C and S100B. These proteins typically undergo a Calcium-dependent conformational change which opens a target binding site. EF-hand proteins involved in buffering/transport include calbindin D9k. These do not undergo Calcium-dependent conformational changes [38,39]**.**

**Figure 3:**
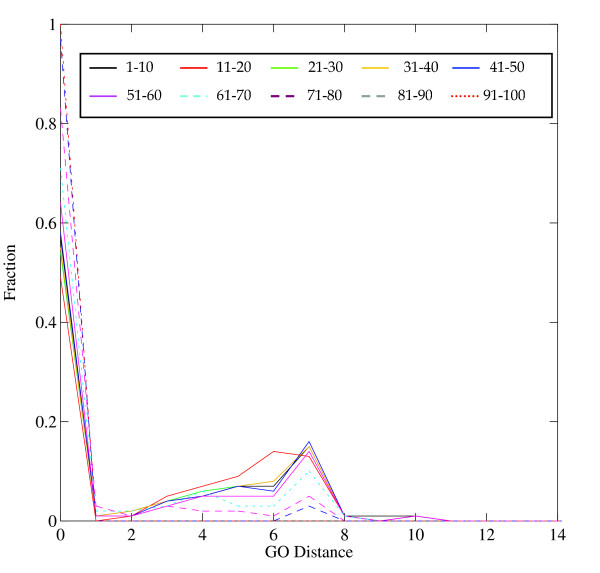
Distribution of Similar functions in the EF-hand family. Figures 3-5 show that the dependence of function divergence on sequence divergence for the EF-hand family. Sequence similarities, measured by the % identical residues in optimal sequence alignment, were divided into bins of width 10%, plotted in different colors as shown in the graph. Abscissa: GO Distance; Ordinate: fraction of comparisons.

**Figure 4:**
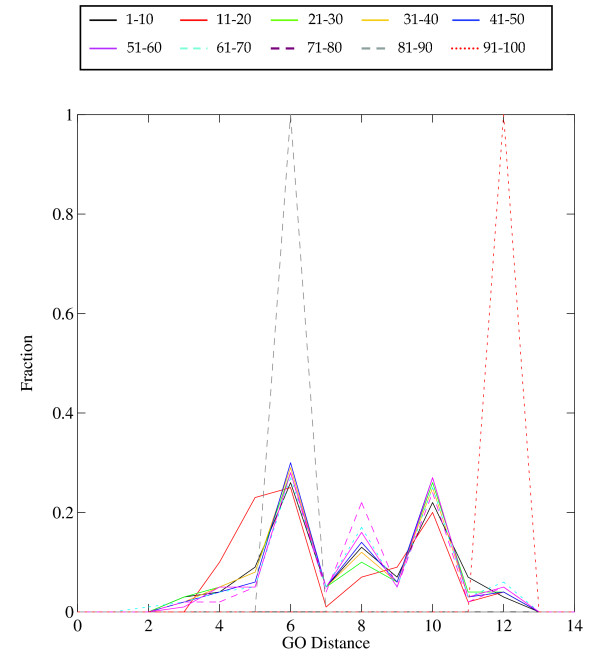
Distribution of Dissimilar functions in the EF-hand family.

**Figure 5:**
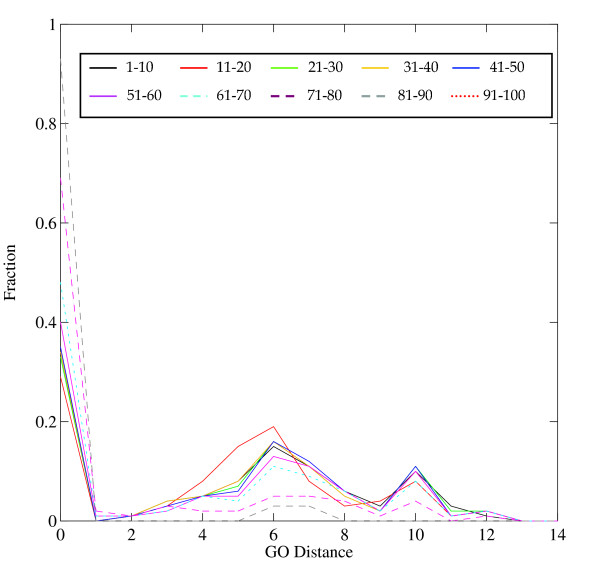
Distribution of Similar + Dissimilar functions in the EF-hand family.

Figures 3, 4, 5 show normalized distributions of functional divergence of pairs of proteins in the EF-hand family, as a function of sequence divergence. The % identical residues in aligned pairs of sequences ranged from 0% to 100%. Pairs of sequences were divided into bins of width 10% sequence identity. The functional distances range between 0 and 12.

#### Similar and dissimilar functions have different distributions

Similar functions (Figure 3) show a dominant peak at distance = 0 (that is, identical function), and a subsidiary peak at 7. It is interesting that the different bins of sequence identity show distributions of similar shape. What distinguishes the distribution of pairs of closely-related proteins from pairs of distantly-related proteins is *not *so much a progressive increase in the set of functional distances represented, but a decrease in the number of pairs with identical function. The distribution (Figure 4) of dissimilar functions of course excludes the peaks at functional distance zero or one, and shows an uneven distribution with peaks between 6 and 10, with a very few pairs at a GO distance of 12. The high spikes are the artifacts of the normalization, in cases where there are very few data. There is a high peak at functional distance 6 for pairs of proteins with 80–100% sequence identity, signifying either recruitment or incomplete annotation (or both).

**Figure 6:**
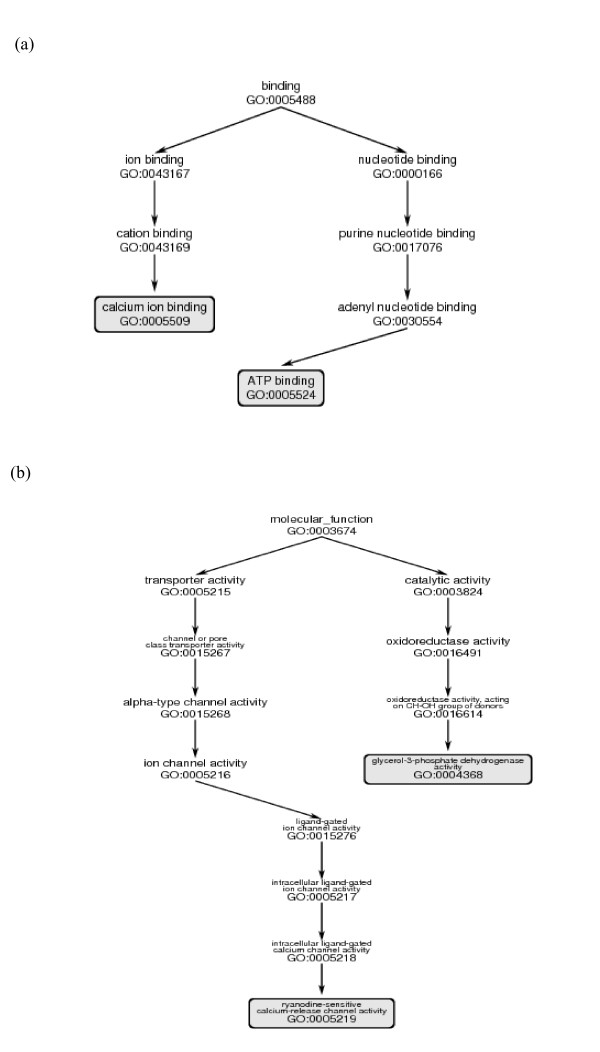
(a) Path in GO DAG between two annotations of proteins of the EF-hand family with Similar functions corresponding to GO distance = 7. (b) Path in GO DAG between two annotations of proteins of the EF-hand family with Dissimilar functions corresponding to GO distance = 12.

Figure 6 shows fragments of the Molecular Function GO DAG containing minimal-length paths between examples of GO IDs corresponding to (a) annotations of EF-hand proteins of similar functions with distance 7, and (b) annotations of EF-hand proteins of dissimilar functions with distance 12.

The graph combining all similar and dissimilar functions (Figure 5) showed three distinct peaks, at 0, 6, and 10; the peaks at 6 and 10 reflecting dissimilar functions. Two factors contribute to the non-smoothness of the distribution: (1) proteins in the EF-hand family are annotated by a relatively small set of distal GO IDs, and (2) there is an uneven distribution of pairs of proteins with different degrees of sequence similarity. The high peaks at 6 and 10 in Figure 6 arise from pairs of protein with 0–10% sequence identity. (The peak at distance 7, prominent in figure 3, is less prominent in Figure 5 because there are many fewer pairs of similar than the dissimilar functions.)

The results show several regularities:

(1) As the sequences progressively diverge, there is a systematic decrease in the number of pairs with distance 0 (identical function) (see Table 1). The % of pairs with 0 distance is approximately constant (about 35%) for bins of sequence similarity < 40%, and then increases sharply. The distribution of similar functions in pairs of proteins with 80–100% sequence identity has a unique peak at 0.

**Table 1:** The increase in percentage of similar function with increase in sequence similarity in the experimental data of EF-hand family. Right column: Left column:

Sequence identity	fraction of comparison with similar functions.
0–10	0.34
11–20	0.34
21–30	0.35
31–40	0.36
41–50	0.41
51–60	0.70
61–70	0.83

> 70% data not available.

(2) The data suggest the interesting result that there is a threshold at about 40% sequence identity, at which the observed behavior changes. For pairs of proteins with 0–40% residue identity, the distribution is largely independent of sequence identity. Above 40% sequence identity, there is a significant increase in similar functions over dissimilar ones. These results, shown in Figure 5 for the EF-hand family, were also observed when all the PFAM data were combined and the contribution of dissimilar functions to each range of sequence identity calculated.

### 2.2 Combined PFAM data

**Figure 7:**
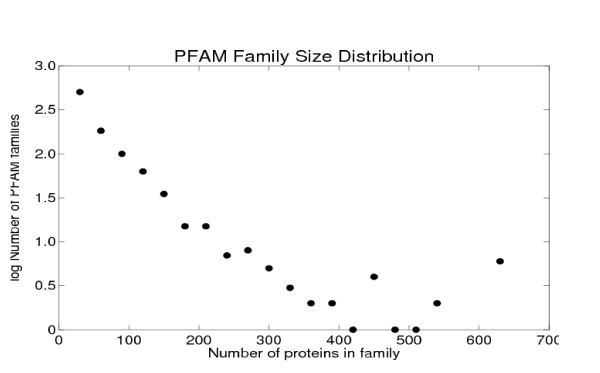
Distribution of sizes of PFAM families.

**Figure 8:**
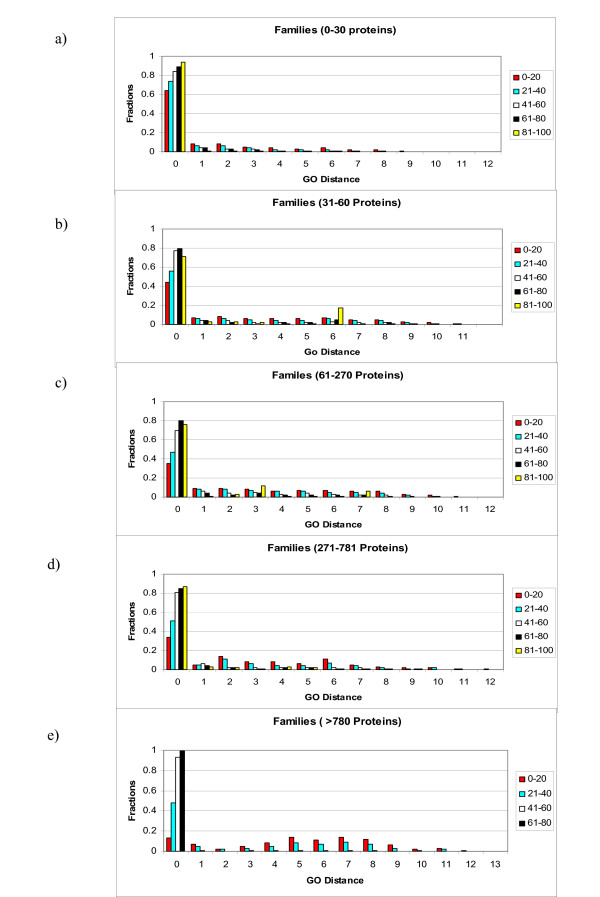
Distribution of functional distances (Y-axis in fraction) in bins of 20% sequence identity (X-axis). The graphs present the distribution of all functions (Similar + Dissimilar).

The number of proteins/PFAM family varied from 2 to 1200 for the collected data (Figure 7). Because of the effects of different sample sizes on the statistics of the distributions, we divided the PFAM families into five categories according to size: 2–30 members (5834 families), 31–60 members (719 families), 61–270 members (244 families), 271–780 members (27), and > 780 members (3 families). Figure 8 shows the relationships between sequence divergence and functional difference for these classes separately, in each case separating ranges of sequence identity into bins of width 20% sequence identity.

Figure 8 shows quantitatively how the distribution of functional divergence depends on the divergence of sequence. For example, Figure 8b describes PFAM families containing between 31 and 60 proteins. The data show generally that as the sequence identity decreases, the percentage of non-identical functions (distances > 0) increases. This graph also contains an example of recruitment (the peak at distance = 6, for the proteins with 81–100% sequence similarity). For proteins with 81–100% sequence identity, 15% of the comparisons have a distance of 6.

The data shown in Figure 8 confirm an "action zone" between 40% sequence identity and 60% sequence identity. This range of sequence identities shows the highest change in the identical functions (GO distance = 0). This suggests a threshold in the behavior: sequence divergence below 50–60% residue identity "releases" function to diverge, or alternatively, functional divergence by mechanisms other than recruitment generally requires > 40% amino acid substitution. Pairs of proteins with all values of sequence identity > 60% had nearly the same contribution from dissimilar functions. This means that for pairs of proteins with > 60% residue identity the fraction of dissimilar functions did not vary strongly with sequence divergence. A similar threshold behavior appears in the relationship between percentage of dissimilar functions and sequence identity. There is a steep increase in the percentage of dissimilar functions as the sequence identity fell below 40%.

**Figure 9:**
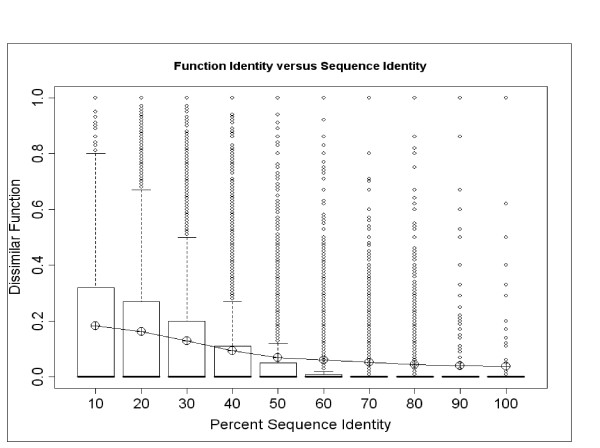
Distribution of fraction of Dissimilar function (Ordinate: fraction) versus sequence identity (X-axis in bins of 10%). The top of each box is the upper 75^th ^percentile, the bottom is the lower 25^th ^percentile. The median of each box is also shown but is superimposed on the 25^th ^percentile. The circles are single extreme cases. The line joins the mean fraction of Dissimilar function at each level of sequence identity. The mean is well above the median due to the extreme skewness of the distribution towards mostly similar function.

To study the variation between different PFAM families, we did separate calculations for each PFAM family and compared results. We calculated the percentage of dissimilar functions within each PFAM family and divided the pairs of proteins into bins of width 10% sequence identity. The results are shown in Figure 9. The data show a very wide overall divergence among different PFAM families in all ranges of sequence similarity. Each column contains data in the full range from 0 to 1 of fraction of dissimilar functions with in a family. However, it is also clear that there is a sharpening of the distribution, a decrease in the mean (corresponding to a greater fraction of similar functions) and fewer outliers, as sequence similarity increases.

Figure 9 shows a systematic difference between residue identities in the ranges 0–50% and 50–100%. Between 0–50% there is a linear decrease in the value of the mean, and almost no further change between 50–100%. This is consistent with a threshold of behavior change between 40–60% sequence identities. The data in the mid quartiles (25 – 75%) also decreases with increase in sequence identity, showing that most of the data are zeros and the number of outliers is also decreasing with the increase in sequence identity.

### 2.3 Comparison with sequence-function correlation based on the Enzyme Commission classification

Other investigators have studied the relation of divergence of function based on the EC classification. Of course, these studies were limited to proteins with enzymatic functions. In a result typical of these studies, Wilson et al. reported a threshold at 30 – 40% sequence identity for onset of more prevalent function divergence in their comparison of sequence and function conservation using Enzyme Commission classification (See ref 33, Figure 7A and 7D).

### 2.4 Comparison of experimental and non-experimental annotations

It could be argued that for purposes of judging the reliability of transfer of annotation among homologous proteins, the comparisons of annotations described in the previous sections are flawed, because the data contain annotations produced by transfer. In order to explore this, we did separate calculations limited to experimentally-based annotations.

GO provides for recording the source of functional assignments, which may be experimental or inferred. The GO consortium classified possible sources of annotation, and ranked them according to suggested reliability. The most direct evidence is experimental: evidence codes TAS (Traceable Author Statement), IDA (Inferred from Direct Assay), IMP (Inferred from Mutant Phenotype), IGI (Inferred from Genetic Interaction) and IPI (Inferred from Physical Interaction). Less direct sources of annotation are ISS (Inferred from Sequence Similarity), IEA (Inferred from Electronic Annotation) and NAS (Non-Traceable Author Statement). We used these evidence codes to compare sequence-function relationships for experimental and non-experimental annotations of proteins.

We extracted proteins of the EF-hand family for which all annotations had experimental support only. This reduced the number of proteins from 498 to 47 (9.5%). We formed two mutually exclusive sets: (1) Proteins with only experimentally verified annotations, and (2) Proteins with no experimentally verified annotations. We collected all the GO IDs for the proteins from both sets and determined the common and different terms. The experimental-based set had 30 unique annotations and the non-experimental set had 65 unique annotations. Both sets of annotations varied from very specific to quite general functions.

**Figure 10:**
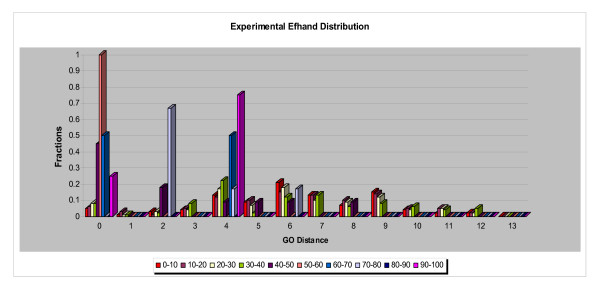
The dependence of function divergence on sequence divergence for the EF-hand family in which the proteins with only the *experimentally *supported annotations were utilized. Abscissa: GO Distance; Ordinate: fraction of comparisons. Different colors show distributions of sets of pairs of proteins with different ranges of sequence similarity, divided into ranges of width 10% residue identity.

**Figure 11:**
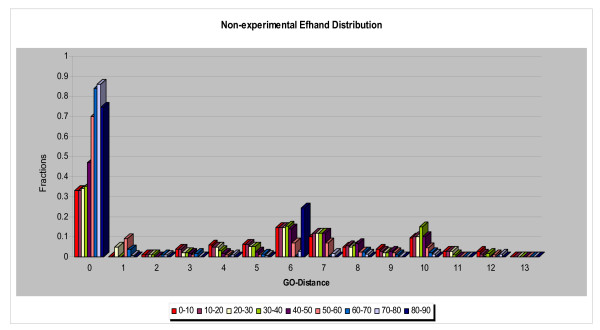
The dependence of function divergence on sequence divergence for the EF-hand family in which the proteins with only the *non-experimentally *supported annotations were utilized. Abscissa; GO Distance; Ordinate; fraction of comparisons. Different colors show distributions of sets of pairs of proteins with different ranges of sequence similarity, divided into bins of width 10% residue identity.

Some of the results that emerged from studying the relationships between the annotation sets were anticipated. All the experimental GO IDs appeared in the non-experimental set, as would be expected if the experimental information "seeded" the annotation of other proteins via transfer of annotations. The comparison of experimental and non-experimental sets also revealed that the percentage of dissimilar functions was higher in the experimental set (~68%) than in the non-experimental set (~40%) for the entire range of sequence similarity. When we compared the normalized data (Figures 10, 11), we observed that (1) a smaller percentage of protein pairs in the experimental set had identical functions (GO distance 0) and (2) comparison of the distributions of functional difference for different ranges of sequence divergence were more similar for the annotations based on non-experimental data than for the annotations based on experimental data.

However, we also observed, to our surprise, that:

1. Many GO IDs in the non-experimental set did *not *appear in the experimental set. This raises the question of what these annotations were based on.

2. The set of non-experimentally-based annotations included *more precise *functions than the experimental set.

For instance, there is solid experimental evidence that proteins of the EF-hand families bind Calcium and Zinc; however, some proteins of the EF-hand family are annotated as binding Magnesium and Iron. The Magnesium and Iron binding annotations are given the evidence code IEA (= Inferred from electronic annotation). Although the non-experimental and experimental annotations share the idea of cation binding, the details – the identity of the metals – are different. Moreover, the non-experimental annotations include specific ligands for which experimental evidence has not been attributed to homologues.

For another example, the non-experimental set contained a GO ID which did not appear with experimental support as the annotation of any homologue: peptidyl-prolyl cis-trans isomerase activity (GO:0003755). The protein FKBP9_MOUSE is annotated with this function with the evidence code IEA. However, a literature search revealed that Shadidy *et al*. reported in 1999 that FKBP9_MOUSE contains an EF-hand domain and showed experimentally-measured peptidyl-prolyl cis-trans isomerase activity [40]. The annotation correctly assigned the function but did not report that the assignment was grounded in experimental evidence.

Such observations persuaded us to leave the experimental/non-experimental comparison at the qualitative level. We conclude that the results of Figure 8 based on a mixture of experimental and non-experimental annotations: (1) probably underestimate, to some extent, the extent of divergence of protein function as a function of amino acid sequence divergence, and (2) probably overestimate, to some extent, the danger of introduction of error in annotation transfer.

## 3. Conclusion

(1) Available data permit a quantitative study of the relation between divergence of sequence and divergence of function in proteins, based on the Gene Ontology functional classification.

(2) Sequence divergence is generally accompanied by higher likelihood of divergence in function, although the phenomenon of recruitment provides exceptions in which proteins of similar sequence can perform very different functions.

(3) There is a threshold at about 50% sequence similarity below which function divergence is enhanced. This is consistent with the conclusions of the previous authors, who used the EC functional classification.

(4) If we were given only the amino acid sequence of a protein of unknown function, and asked to estimate the probability that transferring annotation from the closest homologue in the databanks would not lead to annotation errors, we would base the answer on the distribution of similar and dissimilar functions in homologous proteins only. The variation among different families suggests that it is worth looking at the families individually. This is consistent with the conclusions of Ranea *et al*. [41], who also observed that families evolve at different rates depending on their functional class.

(5) Databases are prone to error, because the recording of experimental sources of functional annotation is a labor-intensive human activity, and because once introduced, errors tend to propagate. Given the very crucial importance of annotation in biomedical research, the development of objective methods for quality control and correction of annotations in databases have been recognized as essential [25].

## 4. Methods

### Sources of data

We downloaded the Gene Ontology network file from the GO consortium website [44] and PFAM domains from the Washington University, St Louis, PFAM server. The March 2005 release of PFAM contained 7868 protein families. PFAM contains seed and full alignments of proteins in each family. We used the seed alignments, which are high-quality alignments that do not change substantially between releases. PFAM uses these alignments as the basis for doing full alignments for the respective PFAM families [42,43].

The PIR database at Georgetown University provided the GO IDs for each protein. PIR presents GO IDs in Molecular Function, Biological Process and Cellular Component categories. We used only the Molecular Function assignments.

### Preprocessing

For each protein we identified the distal GO ID(s) in its annotation set. A distal GO ID is the GO ID included in the annotation of the protein, for which no more specific (descendant) GO ID is part of the annotation of the same protein. For example, suppose that in some data base a protein is given as its functional annotation three GO IDs: 00016788 (hydrolase activity, acting on ester bonds), 0004320 (oleyl-[acyl-carrier protein] hydrolase activity), and 0000036 (acyl carrier activity) (see Figure 3). GO ID 0004320 is a descendant of 0016788. In this chain of descent in the GO graph, 0004320 is the more precise annotation, distal to 0016788. However, 0000036 is not in the same chain of descent. From these annotations we would retain only GO: 0004320 and GO:0000036 as distal GO IDs.

As working data then, for every protein domain we had an amino acid sequence (from PFAM), and the distal GO ID(s) (from PIR). For each pair of proteins in each family, we measured the similarity of the sequence and function.

### Calculation of sequence similarity

We aligned amino acid sequences by the standard dynamic programming algorithm using the BLOSUM 62 matrix [45]. We used the alignment program MUSCLE [46], with default gap weighting. A comparison of sequence similarities computed from MUSCLE pairwise alignments with values computed from the alignments inherent in PFAM showed that the differences were generally so small as to make no significant differences in the results presented (See additional file 1). We note that the pairwise sequence alignment approach provides a measure of sequence similarity that is stable and independent of any realignment or reclassification that PFAM may adopt.

### Calculation of functional similarity

We represented the functional divergence of the two proteins by the distance between their sets of annotations; that is, between the sets of distal GO IDs assigned to each protein. The measure of the distance between sets of annotations was based on a measure of the distance between individual GO IDs. We defined the distance between two *individual *GO IDs as the number of edges in a minimal-length path between the two nodes in the GO DAG that passes through the lowest common ancestor of the two nodes. Based on this, we needed to define a measure of distance between *sets *of distal GO IDs, as might appear in the annotation of a protein with multiple functions.

**Figure 12:**
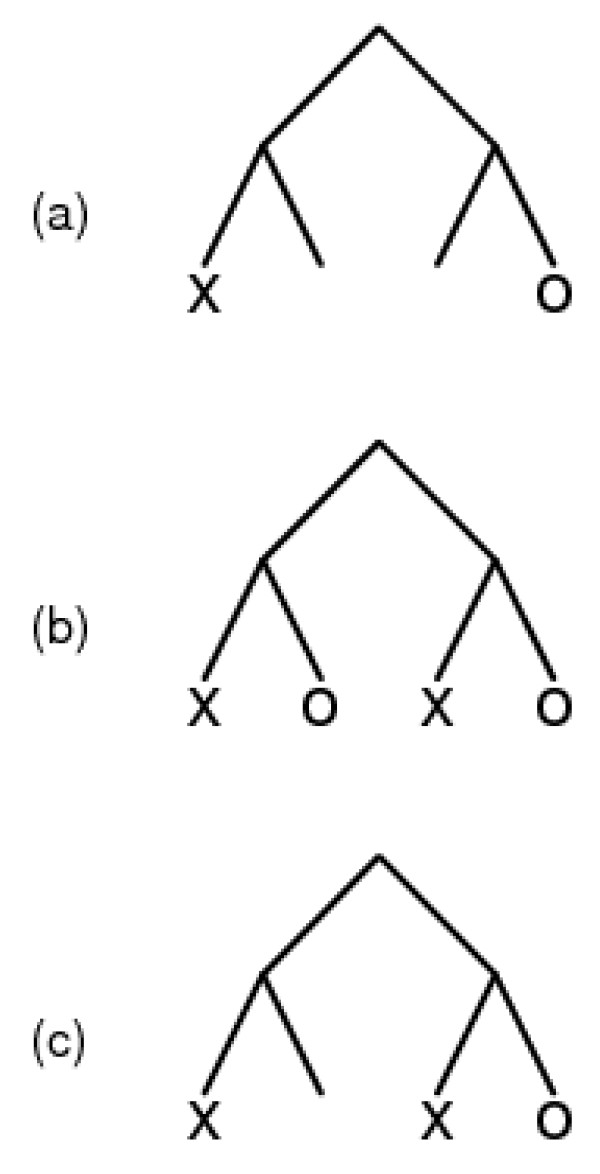
Possible relationships within the GO DAG between functions of two hypothetical homologous proteins, annotated by X and O respectively.

There are several possible ways to assign a metric to the GO DAG. Because there is no natural metric, we have chosen one that appeared suitable after being guided by consideration of specific simple cases (see Figure 12). In each case we are comparing one protein, with one or more annotations labeled X, to another protein with one or more annotations labeled O.

Case (a) is the simplest: each protein has one annotation and the minimum path length between them has length 4. The distance function should report this value.

In case (b), each protein has two annotated functions, appearing on two branches of the graph. However, the two proteins show similarity of both functions: for each X there is an O at distance 2. Although the distance between the leftmost X and the rightmost O is 4, it does not seem reasonable to report a functional distance of 4 between these two proteins. Therefore it would not be suitable to define a distance function as the set of minimal path lengths between every X, O pair.

In case (c), one protein has two annotated functions but the other has only one. The proteins share a similar function: the rightmost X and the O have distance 2. However, in this case, compared to (b), it is relevant that the distance from the leftmost node labeled X to the closest node labeled O is 4. This is a genuine difference between the annotated functions of the two proteins. It may be that protein X was recruited for a novel function not similar to the function of protein O. It is also possible that protein O shares the other function with protein X but is not so annotated. In any event, the distance function should report both 4 and 2. This implies that it would not be suitable to define a distance function as the minimum X-O distance for all X-O pairs.

We therefore adopted the following definition of the difference in functional annotations between two proteins, X and O. For each distal GO ID X, we determine the minimum distance to all the distal GO IDs O, and for each distal GO ID O, we determine the minimum distance to all the distal GO IDs X. This set of values represents the distance between the annotation sets X and O. In the cases shown in Figure 12, the distances reported would be: (a) 4, (b) 2, 2, (c) 2, 2, 4.

Any classification scheme may vary in the fineness with which it distinguishes different regions of its domain. Because for protein function (unlike for sequence or structure) there is no natural metric, there is no direct way to calibrate distances between nodes in either the EC or GO classifications. The problem is somewhat more acute for the GO classification because of the variable depths of the DAG. We explored the possibility of "normalizing" the GO distances according to the local depth of the DAG, but were unable to do this in a consistent way, largely because of the non-uniqueness of the lengths of the paths from any node up to the root.

In order to demonstrate that the problem will not seriously affect the results in at least most cases, we did the following calculation: For all distal nodes in the GO DAG (that is, all nodes that had no lower nodes = nodes farther from the root) we determined the minimal-length path from the root to the distal node. The result was that for 85% of the distal nodes, the minimal path lengths to the root were between 4 and 6. For these cases any reasonable "normalization factor" will vary only between 0.8 and 1.2. Nevertheless, for the particular application: "Given a novel sequence, what is the likelihood of error in transferring annotation between homologues?" we explicitly recommend a homologous-family-by-homologous family approach, in which one would in most cases be comparing functions in similar sections of the GO DAG. For these, the fineness of the distinctions between related functions would be comparable, and the differences in overall depths of different nodes would be a controlled quantity.

## Authors' contributions

All authors together conceived and designed the experiments, analyzed the data, and wrote the paper, with VS taking the leading role. VS and AML wrote required software and ran the computations.

## Supplementary Material

Additional file 1 Comparison of sequence identity between the pair-wise 'Muscle' alignments and the multiple sequence alignments provided by PFAM. The data provides a comparison of sequence identity between the pair-wise 'Muscle' alignments and PFAM alignments. Click here for file
