# Supplementary material for: Quantitative sequence-function relationships in proteins based on gene ontology
Source: BMC Bioinformatics. 2007 Aug 8;8:294. doi: 10.1186/1471-2105-8-294 (PMC1976327; doi:10.1186/1471-2105-8-294)
Supplement: Additional file 1 — Comparison of sequence identity between the pair-wise 'Muscle' alignments and the multiple sequence alignments provided by PFAM. The data provides a comparison of sequence identity between the pair-wise 'Muscle' alignments and PFAM alignments. [file 1471-2105-8-294-S1.doc]

Title: Comparison of sequence identity using the pair-wise ‘Muscle’ alignments and the multiple sequence alignments provided by PFAM.
